# ‘From that time onwards my role changed’. Disclosing suicidality in Australian workplaces a qualitative study

**DOI:** 10.1093/heapro/daaf017

**Published:** 2025-04-10

**Authors:** Martina Odette McGrath, Karl Andriessen, Karolina Krysinska, Nicola Reavley, Jane Pirkis

**Affiliations:** Centre for Mental Health and Community Wellbeing, Melbourne School of Population and Global Health, The University of Melbourne, Level 4, 207 Bouverie Street, Carlton, VIC 3053Australia; Centre for Mental Health and Community Wellbeing, Melbourne School of Population and Global Health, The University of Melbourne, Level 4, 207 Bouverie Street, Carlton, VIC 3053Australia; Centre for Mental Health and Community Wellbeing, Melbourne School of Population and Global Health, The University of Melbourne, Level 4, 207 Bouverie Street, Carlton, VIC 3053Australia; Centre for Mental Health and Community Wellbeing, Melbourne School of Population and Global Health, The University of Melbourne, Level 4, 207 Bouverie Street, Carlton, VIC 3053Australia; Centre for Mental Health and Community Wellbeing, Melbourne School of Population and Global Health, The University of Melbourne, Level 4, 207 Bouverie Street, Carlton, VIC 3053Australia

**Keywords:** suicidality, disclosure, workplace, stigma, compassion, lived experience, social identity

## Abstract

Individuals experiencing suicidality at work may face complex disclosure decisions, involving assessing the risks versus benefits of disclosure or non-disclosure. This study aimed to identify barriers and enablers to disclosing suicidality in workplaces and to explore the responses, support, and accommodation needs for workers experiencing suicidality. We conducted semi-structured interviews with a purposive sample of 30 working adults who reported experiencing suicidality at work and may or may not have disclosed. Using reflexive thematic analysis, we constructed four themes: (i) stigma and discrimination are fears realized, (ii) leaders should address psychosocial hazards, (iii) there’s a price to pay when considering revealing and protecting social identities, and (iv) having safe people and safe systems would help. The study findings indicate that creating more compassionate and empathetically supportive workplaces may help address some of the barriers to disclosure of suicidality, including potentially decreasing stigma and discrimination and addressing psychosocial hazards that act as barriers to disclosure. Considering the role of identity by applying a socioecological lens that includes understanding the role of social identity, belongingness, culture, and marginalization may help to increase understanding of suicidality disclosure decision-making in workplaces. The findings further indicate a need to implement multi-layered systems-level approaches for workplaces to be better equipped to support workers who experience suicidality.

**Contribution to Health Promotion**

- This study presents emerging evidence on an understudied topic, namely, disclosure of suicidality in workplaces.
- Study findings may contribute to identifying opportunities for addressing workplace stigma and discrimination by implementing compassion and empathy-focused strategies and interventions that help respond and support workers disclosing experiencing suicidality at work.
- The findings may extend the understanding about the role of social identities for workers, particularly when they consider disclosing suicidality while working.
- The findings may contribute to existing evidence about the important role of workplace leaders in helping to create positive workplace cultures that can address psychosocial hazards.

## BACKGROUND

Suicide is a major global public health problem. Over 700,000 people die by suicide annually, and suicide is among the top 20 leading causes of death (World Health Organization 2019, 2022a). It is estimated that for each death by suicide, 20 more people engage in non-fatal suicidal behaviour (World Health Organization 2022a). In Australia, one in six people aged 16–85 years (16.7% or 3.3 million people) experience suicidality in their lifetime and 3.3% (644,600 people) do so in a 12-month period (Australian Bureau of Statistics (2020–2022) 2023). Suicidality is understood as suicidal behaviour and suicidal ideation, including suicidal thoughts and plans (Kinchin and Doran 2017, Milner and LaMontagne 2018, Turecki et al. 2019). Suicidality is associated with intense and intolerable emotional pain (Shneidman 1998). The emotional pain of suicidality and suicide death affects many others, including family members, and friends. The emotional pain of suicidality at work and suicide also affects co-workers and employers (Andriessen et al. 2017, Cerel et al. 2018).

One way of helping to address suicide could include helping to understand suicidality and disclosure in all settings, including in workplaces. People experiencing suicidality may be exposed to social isolation, public and self-stigma, and discrimination (Carpiniello and Pinna 2017, Oexle et al. 2019). As co-conspiring forces, the emotional pain of suicidality combined with a fear of stigma and discrimination can result in individuals avoiding disclosure (Brohan et al. 2012, Reavley et al. 2017, Mayer et al. 2020, Zamir et al. 2022). Disclosure has been defined as the sharing of personal information from one person to another (Chaudoir and Fisher 2010, Brohan et al. 2012, Zamir et al. 2022). Studies have also found that people experiencing suicidality may delay, partially reveal or conceal disclosing suicidality until finding a safe and trusted person (Maple et al. 2020). Helping to develop an understanding of the barriers and enablers to disclosing suicidality may help promote earlier help-seeking and reduce the risk of suicide, including in workplaces (Hallett et al. 2024). Along with contributing to preventing suicide, encouraging disclosure of suicidality while at work may also help individuals to access important social and employment-related supports and may help to alleviate some of the emotional stress associated with concealing and managing a stigmatized identity (Ragins 2008, Jones and King 2013). For example, existing evidence on suicidality and mental health disclosure highlight that disclosure decisions are complex, often involving the individual assessing risks versus benefits of disclosing (Sheehan et al. 2019, Hastuti and Timming 2022, McGrath et al. 2023).

With around 60% of the world’s population in employment, it is likely many individuals may experience suicidality while working (i.e. workplace suicidality) (World Health Organization & International Labour Organisation 2022). In 2019, suicide was the fourth leading cause of death for young people aged 15–29 years and over half of all suicides (58%) occurred before the age of 50 (Greiner and Arensman 2022). Workplace suicidality has significant economic impacts on governments and businesses. In Australia, it is estimated that the economic costs of workplace suicidality are over $6.73 billion (Kinchin and Doran 2017). However, there is limited evidence about the prevalence of suicidality in people in workplaces (Howard and Krannitz 2017, Howard et al. 2021, McGrath et al. 2023). More research is needed to investigate the factors involved in workplace suicidality (Milner et al. 2018, Spencer-Thomas 2022, LaMontagne and King 2023, Teoh et al. 2023).

There is, however, increasing evidence focussing on improving workplace mental health, including the role of psychosocial hazards. In Australia, codes of practice for management of psychosocial hazards have been introduced at the Commonwealth Government, state territory level (Safe Work Australia 2022). Workplace psychosocial hazards, as defined in codes of practice, include: (i) work design, (ii) workplace environmental factors (such as the physical location and conditions of the workplace where work will be conducted), (iii) tools and equipment used to conduct the work, and (iv) workplace interactions or behaviours (Safe Work Australia 2022, Workplace Health and Safety Queensland 2022). Scholars have recently reported there is a need to address workplace psychosocial safety as one way of improving workplace suicide prevention efforts (LaMontagne and King 2023, LaMontagne et al. 2024).

Based on current evidence, workplaces represent an important setting to understand the barriers and enablers to disclosing suicidality at work. Helping to understand suicidality disclosure may also contribute to suicide prevention policy (Potter et al. 2019). This qualitative study aimed to explore barriers and enablers to suicidality disclosure at work and to explore the response, support, and accommodation needs for workers who experience suicidality while working. This study may contribute to what is known about responding to suicidal behaviour in working adults (Reavley et al. 2012, Rangarajan et al. 2020, World Health Organization 2022b).

This study was guided by these research questions:

RQ1: What role do stigma and discrimination play in influencing suicidality disclosure decisions at work?

RQ2: What role do workplace factors such as workplace culture, policies, and procedures and workplace anti-stigma and suicide literacy training play in informing suicidality disclosure and non-disclosure decisions?

RQ3: What role does a worker’s professional role play in influencing suicidality disclosure decisions at work?

RQ4: What is known about the workplace responses, supports, and accommodations for workers disclosing suicidality at work?

## METHODS

### Approach

Our study applied a qualitative reflexive ‘Big Q’ interpretivist thematic analysis approach instrumentally selected to align with the lead author’s positionality including social values of wanting to subjectively engage in sense-making of the research and data (Kidder and Fine 2004, Braun and Clarke 2024). We chose to use Big Q reflexive thematic analysis as it allows for subjectively exploring participants’ lived experiences and sense-making relating to complex social problems (Braun and Clarke 2021a).

M.O.M. identifies as having lived and living experiences of suicidality, including in workplaces. These lived experiences led her to lead this research seeking to understand the barriers and enablers to disclosing suicidality in workplaces. K.A. is a senior social work academic with extensive expertise in qualitative and mixed-methods research involving participants with lived experiences of suicide. K.K. is a highly experienced researcher in the fields of suicide prevention and lived experience. These three researchers met regularly to discuss coding and analysis. The senior researchers N.R. and J.P. are experts in the field of population mental health and suicide prevention research and provided advice on the design and conduct of the study, and contributed to manuscript revisions. The whole team met regularly to ensure consistency throughout the research project.

### Recruitment

We purposively recruited participants from November 2023 to January 2024. The study announcement was disseminated on LinkedIn and via the research team’s networks, e.g. known contacts within the suicide prevention and mental health sectors. Participants had to: (i) be adults aged 18 years and over, (ii) be in paid work in any role and organization in Australia, (iii) have experienced suicidality in a workplace, but not within the last 6 months, and (iv) have no relationship with the authors. Participants may or may not have disclosed their suicidality at work. Potential participants contacted the lead author who provided them with a plain language statement and informed consent form and invited them to an online pre-study meeting to confirm eligibility. Participants were offered a $30 gift voucher. A total of 47 potential participants contacted our team, and 30 participated. Reasons for not participating included being ineligible (*n* = 9) or unavailable (*n* = 5) and making contact after the data collection phase (*n* = 3).

### Data collection

M.O.M. conducted semi-structured individual interviews with the 30 participants via Zoom using an interview guide (Supplementary File S1). The interview guide was piloted with an independent researcher not involved in the study but who identified as having previously experienced suicidality in a workplace. The interviews were recorded, and the audio files were kept for verbatim transcription and analysis purposes. Interviews started with questions about participants’ sociodemographic details and their workplace and occupation when they experienced suicidality and considered disclosing. The remainder of the questions focussed on participants’ views and experiences relating to factors that may have acted as barriers or enablers for suicidality disclosure decision-making. Interviews lasted an average of 66 min (range 37–102 min). Participants had a mean age of 42.2 years and 80% were women. Half of the participants came from Victoria and New South Wales, but other states were also represented. Participants worked in a range of occupations, mostly office-based roles and 86% were in full-time employment. The participants’ characteristics are outlined in Table 1. Occupations have been defined according to occupational groupings listed in the Australian and New Zealand Standard Classification of Occupations (ANZSCO) classification (Australian Bureau of Statistics 2022).

**Table 1.** Sociodemographic characteristics (*N* = 30)

Characteristics	*N* (%)
Age range	26–60
	*M* = 42.2
	SD = 2.95
Gender	
Women	24 (80%)
Men	6 (20%)
Geographical region	
Victoria	10 (33%)
New South Wales	5 (16%)
Queensland	5 (16%)
Australia Capital Territory	4 (13%)
Tasmania	2 (6%)
Western Australia	2 (6%)
South Australia	2 (6%)
Occupations	
Managers	8 (26%)
Professionals	11 (36%)
Technicians/Trades	1 (3%)
Community/Personal Services	6 (20%)
Clerical/Administrative	3 (10%)
Sales	1 (3%)

### Ethical considerations

Acknowledging the risks in conducting research on a sensitive topic, we implemented robust risk management and distress management protocols to protect the wellbeing and rights of participants (World Medical Association 2013). We engaged two psychologists to provide support (if requested) during the recruitment and data collection phases. Ultimately, however, no participants requested psychological support. The study received ethics approval from the Human Research Ethics Committee of The University of Melbourne (October 31, ID number: 2023-25996-46688-4).

### Data analysis

De-identified interview transcripts were exported to NVivo 14 software, used for coding, data management, and data visualizations to assist in generating, conceptualizing, and contextualizing themes. Data were deductively analysed, through a lens of the lead researcher’s own experiences and views combined with knowledge from a literature review (McGrath et al. 2023). Data were also inductively analysed, drawn from participant’s interviews. Data analysis follows the six-step reflexive thematic analysis approach (Braun et al. 2019, Braun and Clarke 2023). M.O.M. led the analysis using a non-linear organic process of data familiarization and exploring codes for semantic and latent meaning (Braun and Clarke 2021a, 2021b). M.O.M. developed a thematic table with K.A. based on initial analysis of two transcripts which were used during discussions with K.A. and K.K. to identify areas worthy of closer interrogation and ongoing review. M.O.M. shared the thematic table and theme extracts with K.A. and K.K. to develop and construct themes. After reading and re-reading the transcripts, M.O.M. began generating initial themes, finding patterns, searching for themes, reviewing and modifying themes, crafting and defining themes, and writing up the thematic findings. To assist with reflexivity and organic coding, M.O.M. also wrote journal memos thus documenting her subjectivity relating to the interviews (Braun and Clarke 2021b).

## RESULTS

### Theme 1: Stigma and discrimination are fears realized

Nearly all the participants talked about stigma and discrimination being of significant concern when they considered disclosing suicidality in the workplace. Participants spoke about needing to assess the potential for negative career impacts. Some spoke about feeling as though everything had changed after they experienced suicidality, took leave, and returned to work. Many participants experienced stigmatizing and discriminatory practices such as feeling labelled, ostracized, being demoted, or made redundant. Others spoke about noticing an immediate shift in how they were treated after they inadvertently disclosed. These experiences of stigma and discrimination influenced participants’ views about whether they would disclose suicidality in the future.

*I wasn’t allowed to talk about what had happened being in the psych ward. So yeah, so it was like putting up barriers. And I was literally ostracized at work.* (P10)

*And before I knew it, out of my mouth came, ’Oh. when I was suicidal, I wasn’t thinking about going and seeing my GP or talking to the EAP. I was thinking about how I was going to kill myself and the impact would have on my family.’ And yeah, there was stunned silence around the table. Not one person asked me how long ago it had been, whether I was okay, whether it was current or not. Um, but from that time onwards my role changed.* (P15)

Many participants reported experiencing a lack of compassion and empathy after disclosing suicidality. They felt that this led to unfair treatment, including a lack of dignity and rights to privacy, and that it increased their suicidal distress and decreased their likelihood of disclosing in the future.

*But yeah, I was actually at work and then the police came to the work to do the welfare check. So, it was quite nasty. Then they, and because I was in such a terrible state, they actually took me with them. Um, and I said to them, ‘I refuse to sit in a paddy wagon in the front of the school’. So, we had to wait and sit for an ambulance, and it was just a horrible time.* (P14)

Most participants who spoke about compassion and empathy as a factor when they were considering disclosing suicidality, went on to say they wished that the initial response to suicidality had been a more humane one. Many of these participants added they felt like no one seemed to notice their struggles. Some participants said perhaps the reason for their suicidality going unnoticed was, at least in part, due to how adept they had become at masking their emotional pain. Some participants thought that it would have helped if suicidality was responded to in the same ways as physical and visible health challenges.

*But, I think if people actually knew, all of the people around them that are living with mental ill health that are coming to work, smashing it, high performing employees, but have not disclosed, that would go a long way.* (P21)

*That it doesn’t look like just one picture, if that makes sense like. I, while I was in that period I was going to work still every day. And there were times where I kind of just sat there wondering why no one noticed that I was dealing with something. But, because I went to work every day and smiled. And yeah. I don’t think people realise that it looks different to different people.* (P19)

Nearly all participants noted a lack of suicide literacy in their workplaces and felt that ultimately this negatively informed their suicidality disclosure decisions. These participants wished that everyone in the workplace is suicide literate to support workers experiencing suicidality at work. Conversely, other participants spoke about their workplaces having appropriate levels of training, tailored to the workplace and workers, which they viewed as contributing to a safer and more open workplace to encourage disclosures.

*But, also coming into a workplace where I could very clearly see that it wasn’t suited to handle these sorts of situations. But, also that majority of the people there didn’t have the skills or the literacy to really address that.* (P19)

*Like the training programs that they had were amazing, right? So, they had the, you know, Mental Health First Aid and we also had vicarious trauma [training], which was great.* (P6)

### Theme 2: Leaders should lead to address psychosocial hazards

Nearly all participants mentioned psychosocial hazards, including the role of workplace culture and leadership which seemed to act as barriers to disclosing suicidality. Most participants spoke about the pivotal role of leadership being a factor when they were weighing up whether to disclose suicidality. Some participants spoke about perceiving their managers had created unsafe, unstable, and toxic workplace cultures, not conducive to disclosing suicidality.

*In that particular workplace, like honestly good leadership. I think just the leadership. And I think for me, if they really do care about their people wellbeing, it was evident from my experience, that the issue was at the leadership level.* (P16)

*The general belief [was] that management wouldn’t be that supportive, or if they wanted or they would be, they would prefer, they would rather cover themselves than to be actually supportive. Because yeah, [..] there was no sort of stable leadership, people didn’t know who to talk to and where to go.* (P28)

Many participants also talked about the workplace culture and workplace bullying which led to them feeling unsafe to disclose suicidality. Some participants talked about going to human resources (HR) to find independent support. These participants said they felt unsupported and gaslit (not believed) for airing concerns relating to interpersonal conflicts and bullying with managers. Participants noted that the conflicts and bullying involving managers and HR staff added to their distress levels and contributed to concealing suicidality. Occasionally, some of these participants also spoke emotionally stating that they felt that being exposed to bullying led to their mental and physical health deteriorating, resulting in suicide attempts and permanent physical health issues. For some, these harmful experiences cemented their views about disclosing suicidality in the future.

*So, I was really badly bullied by six people in the workplace. I remember going to my regional manager about how I was feeling, and that I know this is bullying and it’s not okay. I remember saying to her that ‘I wanna die. This is, like you know, like I know how I am being treated is really unfair. This is how it’s making me feel’. And I was told that’s it’s all in my head.* (P12)

Many participants perceived poor job-related factors, including job design, planning, job stress and strain, job insecurity, and lack of communication between them, management and HR being barriers to disclosing suicidality. Some felt these factors affected their sense of helplessness and the likelihood of disclosing. Some participants talked about a lack of psychological safety related to high workloads (job stress and strain) and working in what they described as high-performance (profit over people) focussed cultures as barriers to disclosing suicidality.

*That was a fixation of that department which I think was linked to performance. I think that was also a big factor, as a sort of manager. You know, start saying, ‘I need to take a mental health day’. You know, definitely couldn’t say, ‘Oh, you know, felt like jumping off a bridge today.* (P26)

### Theme 3: There’s a price to pay when considering revealing and protecting social identities

Many participants spoke about how they viewed their suicidality disclosure decision-making through a prism of weighing up the risks versus benefits to their professional or personal identity. Many participants expressed feeling like they lived complicated, sometimes dual and separate lives, navigating precariously between their professional and personal identity, and spoke about how this impacted their suicidality disclosure decisions at work. Some of them added that wanting to protect their cultural and racial identity was a factor when they considered disclosing suicidality, and said they worried whether their workplace would be able to respond in culturally appropriate and non-stigmatizing ways.

*A lot of it was all very interwoven, so that whole it was hard to sort of separate personal and professional identities there, but at the same time, when looking for a psychologist, I had to make sure that it was not somebody that I was going to end up having to work with down the track.* (P11)

*On top of that like I was the only black person in the entire organization. And so with that, I had a lot of expectations on how I should and shouldn’t behave. So, the last thing I wanted was to kind of feed into any stereotypes that people already have of a black person.* (P19)

Some participants, who also spoke about professional and personal identity being a suicidality disclosure factor, talked about how they chose to prioritize their personal values and beliefs. Other participants who described feeling like they had experienced an identity crisis around the time they were thinking about disclosing, said they regretted disclosing.

*You knew that you shouldn’t have disclosed. And it got to that stage, I was talking to friends and everything like that, because I’m quite open. But and they said, don’t disclose that. Just don’t disclose it.* (P10)

Some participants, when they were thinking about their sense of identity and suicidality disclosure, said they felt as if their strong professional identity and work ethic were paramount when weighing up whether or not to disclose. For these participants, being perceived as a high achiever, and a loyal and competent worker, seemed to create unintended barriers (i.e. the risk of being perceived as less competent), which further complicated suicidality disclosure decisions.

*I was a part of an executive leadership team that was all male, all 20 years older than me. And yeah, it just, there was a sense that if I did disclose that it would then be used as a, ’Oh no, look she’s just being hysterical about this issue,’ you know.* (P21)

*So, once you disclose it, work, you can’t take it back, like you can’t retract sharing at work so, and because I guess I got so much of my self-worth from my job, if that was to crumble …* (P9)

Other participants spoke about how, at the time of their suicidality disclosure decision-making, they were motivated by wanting to protect their personal identity and private life. Some participants perceived their work as being protective, mentally and financially, and provided a sense of purpose and routine. A few of these participants, while feeling suicidal at work, chose to conceal suicidality so they could continue working.

*Because, I also, am on my own with a mortgage, with no backup, no one can pay me anything, and I have to work.* (P20)

*So, I could go to work and immerse myself in something and think about something else. Home life still intruded. But, my work is my respite.* (P15)

However, some participants reflected on their disclosure decisions in terms of wanting to be wholly and authentically acknowledged at work as a person living with or having experienced suicidality. Other participants, particularly those who spoke about previous negative disclosure events at work, said they felt as if those experiences had been formative to their views about current or future disclosures, seemingly helping them to crystallize and reaffirm their own moral compass about what matters most in life and instructive to future decisions to always disclose at work.

*And I said this to every boss that I’ve had since that I will never do anything that is against my values, ever. I will never ask anyone else to do what’s against my values. So that is more important. Jobs are jobs. There’s always work around.* (P6)

*I think though my previous situation has made me a bit scared. It’s also kind of maybe a bit brave in the sense that I don’t really want to hide it. But also, if I’m disclosing this sort of thing, and it gets used against me and then it’s probably not the place I want to be, anyway.* (P19)

### Theme 4: Having safe systems and safe people would help

Most participants spoke about a lack of clear workplace systems, policies, and procedures being barriers to disclosing suicidality. They also thought that even when there were systems in place, they were often hard to find, out of date, or made no mention of how the workplace could support workers experiencing suicidality.

*I contacted EAP and they were like they couldn’t advise either way. And I tried to like, read our policies. I was like, ‘What the fuck happens to me?’ If I say something like literally, I just could not. I’ve read through all our policies. And I just genuinely felt very unsafe to say anything. And, so that that was like a total rigmarole.* (P13)

*I mean, it’s a no brainer, really, that in that situation the least you can do is inform the employee what types of supports they’re entitled to.* (P16)

Most participants spoke about their suicidality disclosure decisions hinging on whether they felt they had access to someone they perceived to be a ‘safe’ person (a confidant) with whom they could confidentially share their suicidal distress.

*So, I actually feel like that disclosure came from a place of trust, trust in that relationship, but absolutely not trust in the workforce, workplace I should say.* (P13)

Many of these participants mentioned their managers and how their disclosure decisions were informed by whether they felt their manager was equipped to respond to suicidality disclosure, compassionately and competently. Most participants felt there had been a lack of good responses and supports which proved to be barriers to disclosing suicidality.

*Remember the person in the room. Like, stop panicking, you know, just because someone comes to you with suicidality doesn’t mean they’re asking you to solve it or get rid of it for them or anything in between. They’re actually just telling you, because they’ve got a burden on their shoulders that they’re not sure what else to do. You need support to go through this messy thing that you’re dealing with, which is, could be suicide.* (P9)

Many participants spoke passionately and felt there had been an over-reliance on the employee assistance program (EAP) as the only support they were offered. They stated that because of this perceived over-reliance on EAP, they would be more likely to conceal suicidality rather than seeking help at work.

*So I don’t really have an identified safe person to go to. I know there’s EAP. We get told about EAP. However, EAP is not, it’s not always what you want. You want someone that kind of understands the role you’re doing and can actually on the ground put things in place.* (P30)

Many participants stated, sometimes emotionally, that workplace policies and procedures in the context of suicidality disclosure should look at a person in suicidal distress, as a human being who is simply experiencing deep emotional pain. Some participants thought that it would help if workplace policies acknowledged that although a worker may be experiencing suicidality, they are not necessarily incapable of working. A few participants highlighted the need for workplaces to strive to be more compassionate and empathetic and support all workers to thrive, including those experiencing suicidality.

*My key message to workplaces would be that just because someone is thinking about ending their lives doesn’t mean that they’re incapable of doing the work that they’re being paid to do, if they are given the right supports.* (P15)

*If you’re going to do it, commit to it. And what I mean by that is, … recognise that there’s no silver bullet, that it’s a whole lot of little things that you put in place and keep putting in place, or keep building on that add up over time. There’s no one quick thing that will fix it all, and make you know, make your organization a safe place. It’s a whole lot of little things, over time.* (P23)

## DISCUSSION

This qualitative study aimed to explore barriers and enablers to suicidality disclosure at work and the response, support, and accommodation needs for workers who experience suicidality while working. To answer the research questions, the study developed four themes: (i) stigma and discrimination are fears realized, (ii) leaders should lead to address psychosocial hazards, (iii) there’s a price to pay when considering revealing and protecting social identities, and (iv) having safe systems and safe people would help.

We found that stigma and discrimination acted as barriers to disclosing suicidality. Participants said that they were isolated, sometimes demoted or made redundant, after experiencing suicidality at work. The finding indicating there is a need for more compassion and empathy in workplaces which may in part be an antidote to addressing stigma and discrimination in workplaces and help to support workers experiencing suicidal distress (Meechan et al. 2022). As described by Meechan et al. (2022), compassion and empathy involve: (i) noticing another person’s emotional state, (ii) empathizing (being able to understand another person’s experience), and (iii) taking action to help the other person improve their situation. There has been increasing evidence focussing on helping to understand and improve workplace wellbeing (Litchfield et al. 2016, Teoh et al. 2022). Despite evidence demonstrating that suicide affects many working-age adults, there is a scarcity of research helping to understand workplace suicide and suicidal distress (Howard and Krannitz 2017, McGrath et al. 2023, LaMontagne et al. 2024).

There is also a strong economic argument for addressing stigma and discrimination in workplaces to help prevent suicide and reduce suicidal distress. We found that stigma and discrimination were significant barriers to disclosing suicidality and impeded help-seeking and help-giving behaviours. Recently, scholars have reported the potential economic benefits for governments and employers for addressing workplace suicide could be in the vicinity of $61.26 million per year (Kinchin and Doran 2017).

Achieving more compassionate and empathetic workplaces may require everyone’s involvement. However, as we found, to help achieve a more compassionate and empathetic workplace, it may require good leadership that focuses on role modelling values of human dignity and doing what is right (Brouwers et al. 2020, Meechan et al. 2022). Providing suicide prevention and mental health literacy training may also contribute towards reducing stigma and discrimination, which we found were important barriers to suicidality disclosures. Our finding relating to a need for everyone in workplaces being trained, is well-established and supported by other studies that have found that suicide prevention and mental health tailored and culturally appropriate training could contribute to mitigating workplace stigma and discrimination (Reavley et al. 2018, Van Laar et al. 2019, Bond et al. 2021, Toth et al. 2023).

Participants’ views reported in theme two focussing on the role of leaders in creating safer workplace cultures helping to address psychosocial hazards is in line with other evidence on the crucial role of leaders who could lead and communicate a culture of safety and trust to promote disclosing suicidality and earlier help-seeking behaviours (Rutherford et al. 2023, Wang et al. 2023). Our finding relating to participants feeling unsupported by HR when they sought independent help relating to workplace bullying and interpersonal conflicts with their managers seems to indicate that alongside workplace managers, HR personnel are another important target for suicide literacy and stigma reduction interventions (Brouwers et al. 2020). These findings seem to also corroborate recommendations that workplaces could adopt a social dialogue participatory approach, led by leaders and involving all workers, to help improve social cohesion and psychosocial safety (International Labour Organisation 2024). The finding relating to psychological safety, bullying, gaslighting, and interpersonal conflicts, which we heard were barriers to disclosing suicidality, supports other studies on the need to mitigate the harms associated with bullying for workers experiencing suicidality (Leach et al. 2017, Thompson and Doran 2024).

In our study, we also found that for some participants, bullying, gaslighting, and conflicts, heightened suicidality, resulting in poorer physical and mental wellbeing. However, workplace bullying and its relationship to suicidality is an understudied topic and more research is needed (Høgh et al. 2021). In line with our findings on the need to address job design and job control factors (one aspect of psychosocial safety) and its affect on disclosure decisions, other studies indicated that addressing working conditions, including job design may help to mitigate some of the risks for workers experiencing suicidality (Almroth et al. 2022, Pirkis et al. 2023).

Theme three’s findings relating to the complexities associated with social identities when considering disclosing suicidality in workplaces helps extend on earlier work that has also focussed on managing invisible, often competing identities while working (Clair et al. 2005). We found that perceived professional and personal identity were significant factors for participants considering disclosing suicidality. Mostly, participants appeared to assess the risks versus benefits relating to how they saw themselves in their profession compared to their personal identity. Participants who felt they had a strong work ethic and tightly held professional identity, said they worried about damaging or losing their professional identity and status, which were further barriers to disclosing. This finding is supported by other scholarly work that has indicated that identity, when combined with a fear of losing one’s professional status, such as being seen as a competent and reliable, is a barrier to disclosure (Roskar et al. 2022).

Several participants feared being socially excluded at work (which for some participants, came to fruition). This finding relating to social exclusion and identity, seems to align with recent research aiming to understand how suicide and identity are interrelated social constructs (Mueller et al. 2021). Similarly, other work has also highlighted that a person’s professional identity, social status, combined with a desire for human agency (e.g. wanting to disclose suicidality), is complicated (Collinson 2016). The notion of emotional labour involved in social identity and suicidality disclosure decisions, raised by some participants, seems to be supported by other evidence on the complicated nexus between concealing and revealing, when considering identity and disclosure decisions (Pachankis 2007, Berkley et al. 2019).

This theme also seems to support calls by leading global health and workplace scholars to apply a socioecological lens to understanding how social identity influences a person’s suicidality disclosure decisions (Standley 2022, World Health Organization & International Labour Organisation 2022). According to these authors, applying a socioecological lens, particularly related to the sense of self and social identity, would involve ensuring all structural and cultural factors affecting suicidal thoughts and behaviour are considered. In theme three, we also found that some participants prioritized personal identity and personal factors when thinking about disclosing. For some, disclosure decisions were based on wanting to be open and authentically seen at work or choosing to conceal as a way of protecting their rights to continue working, and for others because of the protective nature of work emotionally and financially. Our findings relating to the role of personal identity (while managing a concealable and stigmatized identity at work) such as living with suicidality and thinking about disclosure, have received support from other research (Follmer and Jones 2021).

In theme four, having safe systems and safe people being helpful to encourage disclosing, participants spoke about feeling like there was insufficient and inadequate systems-level support (policies and procedures), accommodations, and a lack of safe people being barriers to disclosing suicidal distress. Our findings seem to support a recently published workplace mental health framework. This framework, developed by Australian researchers, also highlights the inextricable link between psychosocial safety and workers’ mental wellbeing, providing timely guidance for workplace leaders and managers, with a model presented focussing on three key interrelated levels of intervention, from promotion to responding and underpinned by the need to ensure workers’ mental wellbeing is protected at work (Deady et al. 2024). Our findings are also in line with other studies indicating there is a need to ensure workers can be supported after disclosing by implementing interventions that could assist workers to recover at work and be able to negotiate with their managers reasonable supports and accommodations (Gray et al. 2019, Murphy et al. 2023). Our findings relating to a need to enhance systems are also evidenced by calls for broad-scale universal government and workplace strategies to help address suicidality (Pirkis et al. 2023, White et al. 2023). Similarly, a recent qualitative study exploring participants’ views on systems-level interventions to support workers impacted by the death of a client or a colleague at work (known as workplace suicide postvention and understood to increase the likelihood of these workers experiencing suicidality), found there was a lack of supportive workplace cultures and systems (Clements et al. 2024).

We also found that many participants reported lacking confidence about not having a trusted person with whom they could disclose suicidality. The need for safe confidants has been reported in other studies, which found that having relational safety and trust could help to facilitate safe disclosures at work (Brohan et al. 2014, Sheehan et al. 2019, Byrne et al. 2022, Gullestrup et al. 2023). This finding relating to providing a supportive response to workers disclosing suicidality seems to further demonstrate the critical role of workplace leaders in helping to encourage safe disclosures by fostering compassionate and caring workplace cultures (Barsade and O’Neil 2016, National Suicide Prevention Taskforce 2020). Notably, as found by other researchers, many participants felt that their managers were simply not equipped to respond or support disclosures (Quinane et al. 2021). This finding seems to highlight that the recipients of disclosure in workplaces (usually line managers) are important targets for interventions, such as suicide prevention training, to strengthen managers’ capabilities and confidence to respond to worker’s suicidality, which has also been recommended in other work on health and mental health disclosure barriers and enablers (Bryan et al. 2018, Reavley et al. 2018, Hastuti and Timming 2021, Toth et al. 2021). An example of an intervention that may help involves a competency framework developed in Ireland, specifically designed to equip managers to respond to suicidality (O’Brien et al. 2021).

## LIMITATIONS

The study has several limitations. The sample included a voluntary, mostly female sample. Self-selection bias may be present; and recruitment was only done through researchers’ contacts and LinkedIn. Interviews were conducted using Zoom which may have excluded people without Internet access or not wanting to participate in online interviews. Most participants worked in public service, public health, or social service not-for-profit sectors. Findings may not be generalizable to other industries and occupations. A strength of this study is that most participants had experienced suicidality at work in the last 1–5 years, indicating the findings are contemporary to existing evidence and practices.

## CONCLUSION

Addressing stigma and discrimination by creating more compassionate and empathetic workplace cultures seems crucial in removing barriers to disclosing suicidality at work. Workplace employers and managers should ensure there are trained ‘safe people’ who can support co-workers experiencing suicidality and to help promote earlier help-seeking. Addressing the barriers to disclosing suicidality will require strong leadership, alongside implementing interventions, such as tailored training programs for managers, suicide literacy training for all employees, and addressing the psychosocial hazards of bullying, gaslighting, and workplace interpersonal relational conflicts. Employers and managers should explore opportunities for improving the systems and supports available to workers experiencing suicidality while working. Striving to grow workplaces that are grounded in compassion and empathy, supporting all workers to thrive, including those experiencing suicidality, will require strong leadership to achieve cultural change and workplace wellbeing. More research is needed to increase understanding about the risks and protective factors for workers experiencing suicidality and considering disclosing across a broad range of industries and occupations. Further research may also identify best practice interventions to support people experiencing suicidal distress and who may need or want to consider disclosing while working.

## Supplementary Material

daaf017_suppl_Supplementary_Files_1

## Data Availability

The datasets, including transcripts and analysis of this study, are not publicly available due to protecting participants’ rights to privacy and avoiding individuals being identifiable. Data will be shared by the corresponding author, pending reasonable requests.
