# Supplementary material for: ‘From that time onwards my role changed’. Disclosing suicidality in Australian workplaces a qualitative study
Source: Health Promot Int. 2025 Apr 10;40(2):daaf017. doi: 10.1093/heapro/daaf017 (PMC11983691; doi:10.1093/heapro/daaf017)
Supplement: daaf017_suppl_Supplementary_Files_1 [file daaf017_suppl_supplementary_files_1.docx]

**Individual semi-strucutred interview guide**

*“From that time onwards my role changed.” Disclosing suicidality in Australian workplaces a qualitative study*

| **Socio-demographics** | |
| --- | --- |
| - In which state or territory do you live - Where do currently work? - What is your employment status (eg; are you working full-time, part-time or casual)? - What is your professional job role eg; are you a baker, a teacher or a health care worker, a construction worker etc? |  |
| **Lead question 1 – stigma and discrimination** | **Probing question** |
| Do you think that stigma and discrimination played any role in your decision to disclose or not disclose suicidality at work? | Can you tell me more about this? |
| **Lead question 2 – workplace factors** | **Probing question** |
| Do you think that there are any workplace factors that may have influended your decision to disclose or not disclose your experiences of suicidality while at work? | For example, did things like:   - workplace culture - policies and procedures or - workplace anti-stigma and suicide literacy training   play any role in informing your decision to disclose or not disclose? |
| **Lead question 3 – worker identity** | **Probing question** |
| Do you think that your worker’s role and professional identity played any role in influencing your decision to disclose or not disclose suicidality while at work? | Can you tell me more about this?  Do you think the the type of work a person does negatively or postiviely affects a person’s suicidality disclosure decisions? |
| **Lead question 4 – workplace responses, supports and accommodations** | **Probing question** |
| Can you tell me about your workplace’s response, supports and accommodations | For example, did the availabilty or a lack of a supportive response and accommodations influence your decision to disclose or not disclose? |
| **Final question – what could be help** |  |
| Can you tell me what you think could help improve the experiences and outcomes for someone like you who might also be considering whether or not to disclose suicidality at work? | For example, based on our discussion so today, is there anything in particular you feel is more or less important when thinking about suicidality disclosure at work? eg;   - is it stigma and/or discrimination, - is it things in the workplace like culture, policies and procedures - is it training - or is it having a supportive manager or co-worker with whom to disclose to? - or the availability of adequate supports and adjustments, that are more or less important to you? |
| **Additional question** |  |
| Is there anything we haven’t discussed today in relation to disclosure of suicidality at work that you would like to tell me? | For example, is there one key message or something think you think would really help encourage safe and supportive suicidality disclosure at work and what would that be? |
